# Is a Bioengineered Heart From Recipient Tissues the Answer to the Shortage of Donors in Heart Transplantation?

**DOI:** 10.7759/cureus.25329

**Published:** 2022-05-25

**Authors:** Md Walid Akram Hussain, Pankaj Garg, John H Yazji, Mohammad Alomari, Emad Alamouti-fard, Ishaq Wadiwala, Samuel Jacob

**Affiliations:** 1 Cardiothoracic Surgery, Mayo Clinic, Jacksonville, USA

**Keywords:** lab-made heart, whole heart engineering, heart bioengineering, growing a heart, recellularization of scaffold, whole-organ engineering, cardiac regeneration, 3d scaffold, cardiac decellularization, organ bioengineering

## Abstract

With the increase in life expectancy worldwide, end-organ failure is becoming more prevalent. In addition, improving post-transplant outcomes has contributed to soaring demand for organs. Unfortunately, thousands have died waiting on the transplant list due to the critical shortage of organs. The success of bioengineered hearts may eventually lead to the production of limitless organs using the patient’s own cells that can be transplanted into them without the need for immunosuppressive medications. Despite being in its infancy, scientists are making tremendous strides in “growing” an artificial heart in the lab. We discuss these processes involved in bioengineering a human-compatible heart in this review. The components of a functional heart must be replicated in a bioengineered heart to make it viable. This review aims to discuss the advances that have already been made and the future challenges of bioengineering a human heart suitable for transplantation.

## Introduction and background

According to reports, currently, 64.34 million people suffer from heart failure worldwide [1]. Furthermore, the number of patients with end-organ heart failure is rising, leading to an all-time high in the number of people waiting for an organ transplant [2]. Several strategies have been devised to increase this strained supply of heart for transplantation, including expanding donor criteria [3], use of advanced perfusion machines such as organ care systems (OCS) to improve viability [4], use of normothermic regional perfusion (NRP) in donor from cardiac death (DCD) hearts, and xenotransplantation. Recently, the focus has shifted to new procedures using regenerative cells, angiogenesis factors, biological matrices, biocompatible synthetic polymers, and online registry systems that utilize bioimplants. These advanced technologies are collectively referred to as tissue engineering [5-8]. Ultimately, the goal is to grow a heart de novo. In addition to the unlimited organ supply, the new organ would be antigenically identical to the recipient as the recipient’s cells would be used, eliminating the need for immunosuppressive agents.

**Figure 1:**
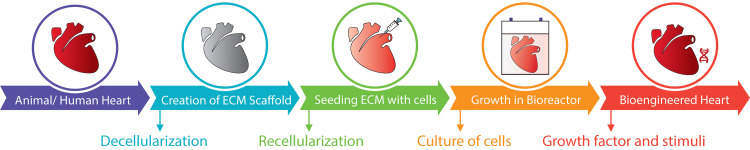
Outline of the processes involved in bioengineering a heart. The figure outlines the process of bioengineering a heart in the lab. The process starts with the decellularization of a human or animal heart which creates a decellularized extracellular matrix (ECM) scaffold. This ECM scaffold is then reseeded with cells (recellularization process) and then cultured in a bioreactor for the growth and migration of cells throughout the ECM with the use of growth factors and various stimuli. This would, hypothetically, create a functioning “bioengineered heart” that can be transplanted into a recipient.

Even though bioengineering a fully functioning heart is in its infancy, huge strides have been made in achieving this goal. Scientists have been able to bioengineer models of the heart, lungs, pancreas, liver, and kidney. An important strategy for supporting the recipient’s cells and creating an autologous tissue/organ is to create a mechanical, geometrical, and biological environment that closely mimics the native organ’s properties. The breakthrough in growing an artificial heart was the invention of the decellularization of extracellular matrix (ECM), which maintains the native vascular network [9]. Numerous tissues and organs have been engineered using decellularization, including livers [10], lungs [11], kidneys [12], corneas [13], bladders [14], vasculature [15], articular cartilage [16], intestines [17], and hearts [18]. There has been some success in engineering a heart in the lab. Although technological innovations and biological model systems have resulted in great progress, constructing such complicated tissue structures effortlessly remains a challenge. This review aims to outline the techniques involved in bioengineering a heart in the lab and the challenges involved in developing it into a viable organ for transplantation (Figure 1).

## Review

Structure of the heart

**Figure 2:**
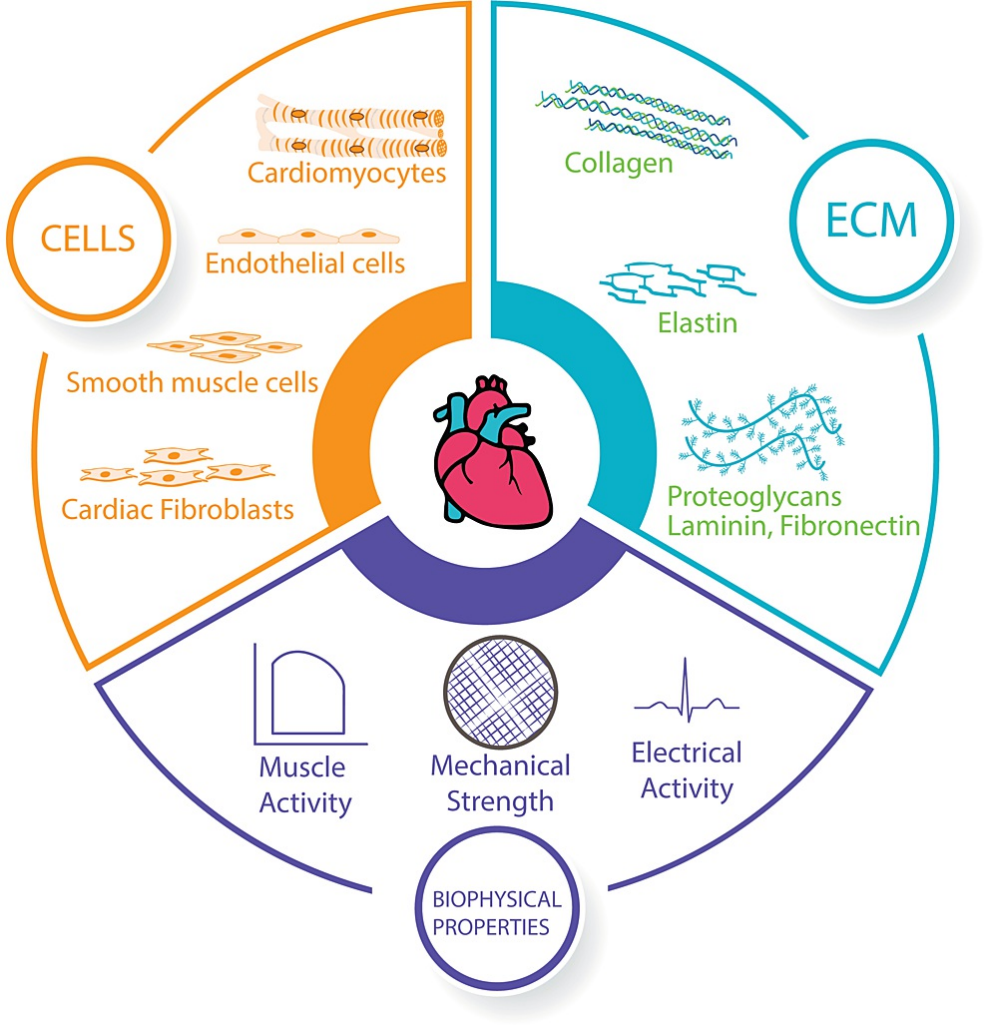
Components of a functional heart. The figure depicts the components of a functional heart. These components can be stratified into three parts. The heart has a myriad of cells. The heart is composed of predominantly cardiomyocytes along with endothelial cells, smooth muscle cells, and cardiac fibroblasts among others. The cells populate a scaffold of extracellular matrix (ECM) which is composed of protein fibers such as collagen and elastin surrounded by proteoglycans, laminins, and fibronectins. This gives the heart its biophysical properties like mechanical strength to undergo rapid muscle movement during the cardiac cycle following an electrical activity. A bioengineered heart must have all three components of the heart to be deemed functional.

The human heart comprises various cells, each specialized to perform a specific task. A human heart contains roughly 2-3 billion cardiomyocytes, making up only about one-third of its total cells [19]. Additionally, other cells include endothelial cells, fibroblasts, and specialized conducting cells like Purkinje fibers. On top of that, structural scaffolds support the functions of cells arranged into structures, such as vessels, muscles, and nerves. These scaffolds mainly consist of polysaccharides and proteoglycans embedded in complex sugars and chemokines matrix, allowing the heart to coordinate its mechanical and electrical functions [20,21]. Sprawled around this is a collection of protein fibers such as collagen and elastin, which confers mechanical strength to the heart and allow for the constant loading and unloading forces [22,23]. Thus, it is necessary to construct a scaffold around which the specialized cells can grow and maintain vitality through blood perfusion to recreate a functioning heart in a laboratory [24] (Figure 2).

Types of ECM scaffolds available

Extracellular matrix (ECM) and cells in an organ display a “dynamic reciprocity,” whereby the ECM constantly adapts to the demands of the cells [25], and selecting the appropriate scaffold is the key component for growing a viable organ in the lab. Researchers have also studied various synthetic scaffolds as potential surrogates for the ECM, but none can replicate its intricacy or structure compared to native ECM. It is possible to “vascularize” synthetic materials such as polylactic acid (PLLA) and polylactic glycolic acid (PLGA) and to produce them consistently [26,27]. The significant advantage of synthetic ECM is its production scalability as it does not require to be harvested from living tissue, but these do not match the native myocardium’s tensile strength. Hydrogels have also been studied extensively and even accepted by the Food and Drug Administration for drug delivery and adjunct for cell therapy. Hydrogels consist of a cross-linked hydrophilic polymer matrix with over 30% water content [28]. However, they have poor cell retention [29] or poor tensile strength [30]; hence, they are not feasible as a primary scaffold for constructing an organ. Decellularizing the whole heart and leaving the ECM serves as a potential solution to this problem with the particular advantage of having a balanced composition of all the proteins present physiologically [31].

Creating the “ideal” scaffold: decellularization of the heart

The Badylak laboratory developed the first technique for decellularizing tissue [32]. This process involved the removal of the cell, leaving only the ECM, which retained its composition, architecture, and mechanical properties. There are several methods for removing cells from the ECM. These methods include physical methods (e.g., freeze/thaw cycles), enzymatic degradation (e.g., trypsin), and removal by using chemicals (e.g., sodium dodecyl sulfate) [33]. Ott et al. noted that decellularization could be achieved with different detergent solutions. Comparative studies on decellularization methods have mixed results regarding the superiority of different techniques [34-37]. Based on the results, the sodium dodecyl sulfate (SDS) solution was found to be the best [18]. However, a few studies have suggested that SDS treatment causes degradation of the ECM with a reduction in elastin, collagen, and glycosaminoglycans (GAG) content [34]. The decellularization process utilizes 1% SDS perfused through the coronary circulation, followed by washing it with de-ionized water and subsequently 1% Triton-X-100 (Sigma). Finally, the organ remnant is washed with phosphate-buffered saline (PBS) wash buffer, antibiotic, and protease, leaving a decellularized ECM [38,39]. Using this technique, they decellularized the heart, reseeded it with neonatal cardiac cells, and grew the first beating rodent heart in the lab [18]. Decellularized tissue provides a dynamic environment for the orientation and coupling of cells and facilitates the exchange of nutrients and oxygen throughout the depth of the tissue. Moreover, this process efficiently removes both allogeneic and xenogeneic antigens, possibly preventing the need for immunosuppressants [33], which is especially important as one of the causes of heart failure in transplanted hearts is myocardial fibrosis from chronic rejection [40]. This process can be potentially avoided by using a decellularized heart to generate an ECM scaffold which can then be repopulated using the recipient’s cells.

Sources for creating ECM scaffolds

Researchers have used animal heart ECM and human heart ECM scaffolds to provide this decellularized ECM scaffold. The porcine heart has often been deemed suitable for its similarity with the human heart [41]. As decellularization removes most of the cells, much of the antigen load is removed. However, the porcine heart ECM contains α-1,3-galactose epitope (α-gal), which can stimulate an immune response [42,43]. One way to circumvent this is to use pigs lacking α-gal epitope, but this technique needs further research. Another possible problem with using a porcine heart is the possible risk of horizontal transmission of porcine viruses like the porcine endogenous retrovirus, cytomegalovirus, HSB, circovirus, etc. [44,45]. Although a few tests can detect the presence of these viruses, they have poor sensitivity, and hence further work has to be done [46].

A cadaveric heart that is unfit for transplant can also be used to harvest an ECM scaffold [47]. The only drawback to this is that it may not always be possible to achieve the desired level of tissue engineering fidelity with these matrices because they may be damaged or diseased. Moreover, there is an assumption that they are superior for the growth and differentiation of human cells, but there is no robust evaluation to support this assumption. The method for decellularization of the cadaveric human heart is similar to that of other animals, utilizing 1% SDS and 1% Triton X-100, with the only difference being a longer perfusion time for these chemicals [48,49].

Recellularization of scaffolds

These cells are highly specialized and terminally differentiated, and hence, they do not proliferate normally. Therefore, to repopulate a human-sized scaffold, autologous human cardioblasts must be isolated or expanded in large quantities. Hence, for the recellularization of ECM, a method of inducing progenitor cells had to be devised. Thus, the discovery of methods to reprogram or induce adult cells into pluripotent stem cells was a significant milestone in stem cell biology and tissue bioengineering [50-52].

Once we have the cells for repopulation of ECM, recellularization is required to achieve a functional organ product for implantation. For recellularization to be achieved, choosing appropriate cell sources, seeding cells optimally, and cultivating them using organ-specific cultures are needed [24]. Cells from fetuses and adults, embryonic stem cells (ESCs), mesenchymal stem cells (MSCs), and induced pluripotent stem cells (iPSCs) have all been used [24]. Obtained with ease and ethically, stem cells from bone marrow stroma or adipose tissue (MSC) have shown promise as the ideal cells for recellularization [53]. In addition, human somatic cells can be reprogrammed to produce iPSCs, and they exhibit properties similar to ESCs [54].

A potential solution to the problem of getting a large number of human cells for tissue engineering or other regenerative medicine approaches is the ability to produce iPSCs from readily available autologous cells such as fibroblasts or blood cells [55,56]. The only drawback to using iPSCs is the possibility of teratoma formation due to its pluripotent nature [48,57]. However, the potential solution to this problem is to allow controlled differentiation toward a cardiac lineage before implantation into the ECM [58]. Although previously any attempts to produce iPSCs would result in karyotype instability [59], recent advances have been made with iPSCs maintaining chromosomal integrity [60]. These advances have ushered a step forward in the pursuit of creating viable organs in the lab.

Cell seeding techniques depend on the type of organ being engineered, and, for the heart, it usually involves seeding by perfusion through the vascular tree [24]. This step is called re-endothelization and is usually the first step to recellularization. A dynamic communication between endothelial cells and cardiomyocyte populations occurs via direct cell interactions and the secretion of various factors [61,62]. It is evident from multiple reports that seeding endothelial cell populations and cardiomyocyte populations simultaneously provides functional benefits that aid in maintaining the recellularization process [63]. Interestingly, endothelial cells have also demonstrated the ability to differentiate into cardiomyocytes in other cardiomyocyte cells [64], which may aid in more efficient recellularization. Moreover, besides the advantage, the recellularization of both the vascular tree and the heart parenchyma must be uniform to prevent two key issues in the heart, namely, thrombogenesis [65] and arrhythmogenesis [66].

Improved cell concentration and diffusion over the scaffold can be achieved by optimizing the mechanical environment, scaffold coating, and cell perfusion systems by using multiple perfusion routes simultaneously, which for the heart involves both direct intramyocardial injections and perfusion of the vascular tree [67]. However, the potential problem with intramyocardial injections is that even though the injection site shows dense cellularity, the cells are generally poorly distributed throughout the scaffold [58]. Moreover, sequential injections of cardiac cells will likely be required to rebuild the chamber parenchyma, which may compromise matrix integrity [48]. Nevertheless, given that cardiac cells include fibroblasts, in which ECM is produced and secreted, there is a possibility that endogenous matrix repair may occur after cell seeding to help resolve this issue [62].

While sourcing cells for recellularization using stem cells is a work in progress, multiple studies have explored ways to develop mature cardiomyocytes derived from iPSCs that are more physiologically similar to native cardiomyocytes [68,69]. One of the most recent cardiac constructs was engineered using PSC-derived cardiac cells in a ratio of equal cardiomyocyte and noncardiomyocyte cells, cultured in serum-free media [70]. Cardiomyocytes cultivated in this method were elongated, had organized sarcomeres and distinguished bands, and exhibited increased contractility [70]. It is encouraging to see these results that stem cells can be used to produce cardiomyocytes similar to native mature cells, reinforcing the notion that stem cells can be a cardiac cell source.

Growing the heart in a bioreactor

After enough cells have been seeded onto an organ scaffold, cell culture is required. A bioreactor is required for perfusion and provides a nutrient-rich environment that encourages organ-specific cell growth [24]. Bioreactors should allow nutrient-rich oxygen to be pumped with adjustable rates of flow and pressure and monitor and control the pH and temperature of the media. Moreover, mechanical stimulation is also an essential component for engineering organs of the musculoskeletal and cardiovascular systems [71]. A wide range of mechanical properties is employed in the design of bioreactors, including substrate stiffness and dynamic changes in stiffness throughout culture, pulsatile flow, and providing stretch to enhance cell maturation, alignment, and generation of force in engineered constructs [72]. Presently, there are several types of bioreactors available, with Radnoti [73] and BIOSTAT B-DCU II [74], to name a few. In addition, there has been an increase in bioreactor designs incorporating real-time monitoring to assess the status of engineered tissues. These designs may incorporate biochemical probes to assess transmural pressure changes or sampling ports to test cells’ viability and biochemical composition after recellularization [75,76]. The incorporation of sampling methods within bioreactor designs will keep constructs sterile, allowing for modifications in stimuli to be made while maintaining a closed system, and providing researchers with valuable feedback on cell responses throughout bioengineering. Further research is being conducted to make bioreactors that can be used to maintain the perfect milieu for growing these bioengineered tissues and organs.

Evaluating the organ for functionality

For an organ to be viable for transplant, three things must be ensured: sterility of the process, structural integrity, and, lastly, patency for surgical anastomosis. Biological tissues are sterilized by gamma radiations or peracetic acid at low concentrations before the ECM is repopulated with cells [77]. Once the cells are added, antibacterial, antifungals, and other antibiotic drugs can be utilized. It is re-evaluated for integrity before the ECM is recellularized and only gets the green light for cell seeding if structural integrity is maintained. Interestingly, with the aid of endoscopy, decellularized constructs can be easily manipulated and visualized for macro and microstructure defects at the level of chambers, papillary muscle, and valves [47]. One of the most important aspects of evaluating the integrity of ECM is to check for intact coronary vasculature, which can be done by micro-optical coherence tomography [48].

Heart constructs engineered in the lab have been demonstrated to undergo cyclical muscular contraction but also have been shown to respond to drugs and exhibit electrical activity. However, electrocardiography analysis of the bioengineered hearts has shown irregular wave morphology due to loss of coupling between cardiomyocytes [78]. Therefore, it will be crucial to develop continuous monitoring of cardiac electrophysiology, function, and even vascular patency if these artificial constructs can be transplanted into patients.

Limitations and future prospects

Over the past decade, research in regenerative medicine has enabled us to understand better the challenges associated with developing a bioartificial heart. The first challenge was creating a biocompatible scaffold which has already been resolved with the development of various decellularization techniques, making it possible to generate an anatomically accurate and vascularized heart scaffold. With the advent of newer techniques for iPSC generation of stable karyotype, cell generation is also potentially resolved. Presently, research has to be aimed to address the challenges in reseeding the ECM scaffold. A potential solution might be the advancement in 3D-printed matrixes with embedded cells. However, decellularized ECM remains the gold standard for now as 3D-printed matrixes cannot replicate the complexity and structural integrity of the natural component of ECM.

Another potential problem is the creation of a bioreactor that can efficiently maintain the environment required for the growth of cardiac and other differentiated cells around the decellularized ECM scaffold. Constructing organs is no easy feat and involves much technical expertise. Hence, many resources are required in every step of artificially reproducing tissues and organs. Thus, even if bioengineering a heart is a possibility in the near future, it may not be financially feasible to use them for transplantation until the cost of making such constructs is lowered. Additionally, we do not know the long-term viability of such constructs. These constructs use chemicals to decellularize ECM as well as induce the conversion of adult cells into pluripotent cells. Some questions arise on how the complex network of cells and ECM would interact over the long run. The heart is a complex organ that requires a highly specialized conduction system to ensure efficient, coordinated, and purposeful contraction of the heart chambers. Any deviance may lead to fatal arrhythmia or thrombus formation. We are yet to reproduce a perfect conduction system in the lab, let alone test its long-term functionality. Furthermore, the use of induced pluripotent cells also raises the prospect of long-term tumorigenesis and malignancy. Despite rapid advances in bioengineering and artificial hearts, research and clinical trials must be conducted to determine the long-term feasibility of using these organs.

## Conclusions

It is an exciting era for biomedical engineering that carries considerable potential to address damaged organs, either via repair or replacement. The advances in heart bioengineering have been astounding. However, further research must be conducted till a mechanically, electrically, and physiologically well-coordinated organ can be constructed and ultimately transplanted into patients needing it. To propel the field forward in the quest for creating unlimited immunotolerant grafts, a coordinated approach should be fostered among researchers, clinicians, regulatory bodies, and society.
